# *Dioscorea* Phytocompounds Enhance Murine Splenocyte Proliferation *Ex Vivo* and Improve Regeneration of Bone Marrow Cells *In Vivo*

**DOI:** 10.1093/ecam/neq032

**Published:** 2011-04-14

**Authors:** Pei-Fen Su, Chin-Jin Li, Chih-Chien Hsu, Spencer Benson, Sheng-Yang Wang, Kandan Aravindaram, Sunney I. Chan, Shih-Hsiung Wu, Feng-Ling Yang, Wen-Ching Huang, Lie-Fen Shyur, Ning-Sun Yang

**Affiliations:** ^1^Agricultural Biotechnology Research Center, Academia Sinica, no. 128, Section 2, Academia Road, Nangang District, Taipei 115, Taiwan; ^2^Department of Medical Research and Education, Taipei Veterans General Hospital, Taipei, Taiwan; ^3^Department of Cell Biology and Molecular Genetics, University of Maryland, MD, USA; ^4^Department of Forestry, College of Agriculture and Natural Resources, National Chung Hsing University, Taichung, Taiwan; ^5^Institute of Chemistry, Academia Sinica, Taipei, Taiwan; ^6^Institute of Biological Chemistry, Academia Sinica, Taipei, Taiwan; ^7^College of Life Science, National Taiwan University, Taipei, Taiwan; ^8^Institute of Life Science, National Central University, Zhongli, Taiwan

## Abstract

Specific cytokines have been tested clinically for immunotherapy of cancers; however, cytotoxicity has often impaired their usefulness. Consequently, alternative approaches are increasingly desirable. *Dioscorea* spp. tuber is a widely used traditional Chinese medicinal herb claimed to confer immunostimulatory activity. In this study, we evaluated *Dioscorea* as an adjuvant therapy for use alongside chemotherapy for cancer. Phytocompounds from *Dioscorea* tubers were ethanol fractioned and used for *ex vivo* splenocyte proliferation assay or *in vivo* force-feeding of mice pre-treated with the chemotherapy agent 5-fluorouracil. Co-treatment with a 50–75% ethanol-partitioned fraction of the tuber extract of *D. batatas* (DsCE-II) and interleukin (IL)-2 resulted in a significantly higher rate of murine splenocyte cell proliferation *ex vivo* than treatment with DsCE-II or IL-2 alone. This DsCE-II fraction, which contains a polysaccharide with a high proportion of **β**-1,4-linkage mannose (≥64%), also promoted the regeneration of specific progenitor cell populations in damaged bone marrow tissues of 5-fluorouracil-treated mice. Colony-forming unit (CFU) analyses demonstrated that the population of CFU-GM cells, but not CFU-GEMM or BFU-E cells, preferentially recovered to *~*67% in the bone marrow of immune-suppressed mice fed with DsCE-II. DsCE-II efficacy level was *~*85% of that obtained by subcutaneous administration of recombinant G-CSF proteins (5 **μ**g kg^−1^) in mice tested in parallel. This study suggests that the DsCE-II fraction of *D. batatas* extract may be considered for further development as a dietary supplement for use alongside chemotherapy during cancer treatment.

## 1. Introduction

Over the last decade, there has been rapid growth in the use of alternative medicines. Natural products, including many plants traditionally used as medicinal herbs, are being re-evaluated as key components in future medicine or nutritional science [1]. Many researchers believe that medicinal botanicals may be useful in regenerative and preventive medicine, especially for tissue-healing and immune-enhancing activities. Recently, however, there has been concern about the safety and effectiveness of these remedies [1–8]. Therefore, systematic rigorous scientific studies of frequently used medicinal or nutritional supplement herbs are urgently needed.

In Asia, *Dioscorea* spp. is a popularly used, traditional Chinese medicinal (TCM) herb that is generally taken alone or in multiple-herb formulations for a range of ailments. Some biological effects of *Dioscorea* spp., including the induction of hypoglycemia in experimental mice and rabbits [9, 10], as well as anti-bacterial [11], antioxidative and hypolipidemic activities [12], have been reported. Anecdotal evidence suggests that *Dioscorea* tubers taken as a food supplement may promote human health by “regulating and upgrading the immune responses” [13]; however, credible experimental data is still lacking.

A fundamental aspect of the immune system is the induction and regulation of the proliferation of specific immune cell populations. The spleen is the major site of immune responses to blood-borne antigens and is also a site of hematopoiesis in rodents [14]. Bone marrow tissues consist of pluripotent hematopoietic stem cells as well as stromal cells which provide delicate environments for growth and development of stem cells [15].

In this study, we used murine splenocytes and bone marrow cell proliferation systems *ex vivo* and *in vivo* to evaluate the bioactivity of a partially purified phytocompound fraction of *Dioscorea* tuber extract on murine immune cell systems. This study aimed to accumulate scientific evidence to evaluate more than 1000 years of use of this traditional herbal medicine as an immune-modulator.

## 2. Methods

### 2.1. Preparation of *Dioscorea* Plant Crude Extracts

**Figure 1:**
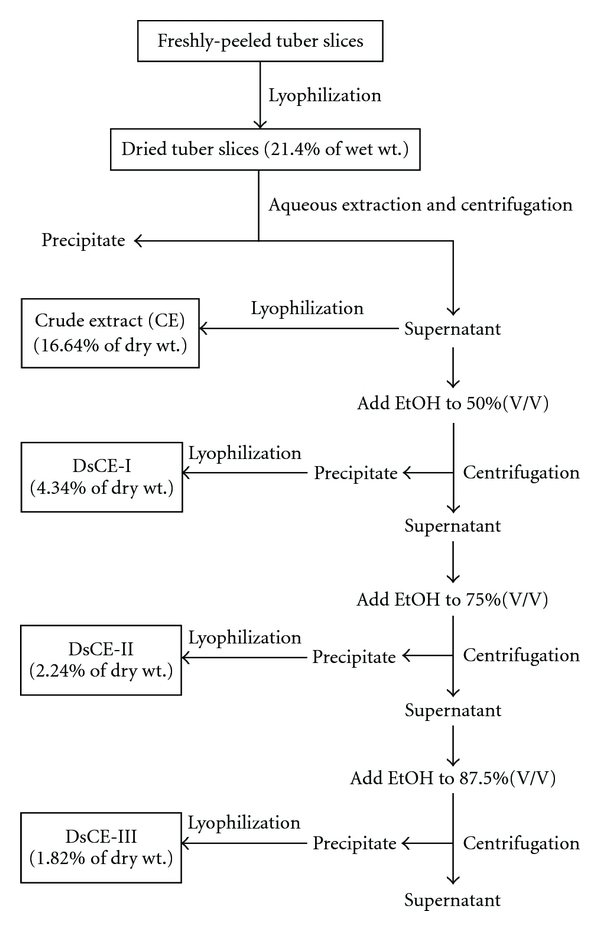
Protocol for preparation and bio-organic fractionation of *Dioscorea* plant extracts. wt., weight.

We used three species [*D. batatas* Decne., *D. alata* L. and *D. pseudojaponica*) and one cultivar, *D. alata* (*D. alata* L. var. *purpurea* (Roxb.) M. Pouch.] of the genus *Dioscorea*. The authenticity of all plant material was validated by Dr Sin-Yie Liu, Taiwan Agricultural Research Institute. Cultivation, growth, taxonomy, and agricultural practice details have been previously reported [16]. *Dioscorea* tubers were peeled, sliced (2–4 mm), lyophilized, and stored in a desiccator at room temperature until use. Dried slices of plant tubers slices were ground in a mortar prior to aqueous extraction. The extraction protocol is shown in Figure 1 In brief, 10 g tuber powder was mixed with 100 mL Milli-Q water, stirred for 1 h at room temperature and centrifuged at 24 000 g for 20 min at 4°C. The supernatant was filtered through glass wool. The pellet was resuspended with another 100 mL water, stirred, centrifuged and re-extracted as above. The supernatants from two extractions were then pooled to yield a crude extract (CE) fraction, with 16.6% dry weight of the original raw materials. The CE fraction was further extracted stepwise with 50, 75 and 87.5% (V/V) ethanol. The ethanol-insoluble fractions were collected by centrifugation at 24 000 g for 20 min at 4°C; the pellet was lyophilized and then dissolved in sterilized water at 10 mg mL^−1^. The fractions were named DsCE-I, DsCE-II and DsCE-III. The yield of DsCE-I, -II and -III was 4.34, 2.24 and 1.82% dry weight, respectively, of the CE. *Limulus* amoebocyte lysate (LAL) assays (Associates of Cape Cod, Falmouth, MA, USA) were performed to detect possible endotoxin contamination. The level of endotoxin found in DsCE-II was lower than 0.04 EU *μ*g^−1^ in each tested preparation.

### 2.2. Fractionation and Characterization of DsCE-II from *D. batatas*

Anion ion exchange column chromatography was used for DsCE-II fractionation. Forty-milligram DsCE-II in 5 mM Na_2_HPO_4_ (pH 7.5) buffer was loaded onto a Q Sepharose Fast Flow ion exchange column (Pharmacia, Stockholm, Sweden) pre-equilibrated with the same buffer. Fractionated DsCE-II was collected from the eluants with a 0–0.75 M NaCl gradient in 5 mM Na_2_HPO_4_ buffer (pH 7.5). The protein and saccharide contents in each fraction were determined with a spectrophotometer by absorbance at A_280_ and the phenol-sulfuric acid method [17], respectively.

### 2.3. Mice

Female BALB/c mice aged 8–12 weeks from the National Laboratory Animal Breeding and Research Center, Taipei, Taiwan, were maintained under standard pathogen-free conditions. All facilities were approved by the Academia Sinica Institutional Animal Care and Utilization Committee (IACUC), and all animal experiments were conducted under the institutional guidelines established by the Animal Core Facility and IACUC in Academia Sinica, Taipei, Taiwan.

### 2.4. Preparation of Splenocytes and Bone Marrow Cells

Murine splenocytes were prepared as previously described [18] with minor modifications. Mouse spleens were surgically removed and minced at room temperature in RPMI 1640 medium. The tissue debris was removed by passing the cell suspension through a cotton column, and erythrocytes were then lyzed by the addition of ACK buffer (150 mM NH_4_Cl, 1.0 mM KHCO_3_, 0.1 mM Na_2_EDTA, pH 7.2). The splenocytes were grown in RPMI 1640 medium supplemented with 10 mM HEPES (pH 7.0), non-essential amino acids, 50 *μ*M *β*-mercaptoethanol, 0.03% l-glutamine, 50 *μ*g mL^−1^ gentamycin and 10% heat-inactivated (56°C, 30 min) fetal bovine serum. Bone marrow cell isolation followed the methods described by Randall [19] and Wlodarski et al. [20].

### 2.5. Cell Proliferation Determination using the H-thymidine Incorporation Assay

Splenocytes (2 × 10^5^ per well) were seeded in a 96-well plate, with a final volume of 200 *μ*L. Herbal extracts or chemicals were added at the indicated concentrations, incubated for 48 h, and then labeled for 16 h with 1 *μ*Ci/well ^3^H-thymidine (NET027X, specific activity 20 Ci mmol^−1^, NEN Life Science, Boston, MA, USA). Concanavalin A (Con A) (1 *μ*g mL^−1^) and water (solvent for *Dioscorea* extract) were applied as positive and negative controls, respectively. Triplicate culture samples were treated at indicated dosage. The labeled cells were harvested with a Cell Harvestor (Packard, Meriden, CT, USA) following the manufacturer's instructions, and radioactivity was determined by TopCount*·*NXT (Packard, Meriden, CT, USA). ^3^H-thymidine incorporation is expressed as the radioactivity in counts per minute per well of the experimental group minus the counts per minute from the negative control (medium only) set.

### 2.6. Treatment of Mice with 5-FU and Herb Extracts

5-FU was used to depress murine hematopoietic cells as described by Wlodarski et al. [20] and Cao et al. [21]. Female BALB/c mice were divided into groups of three animals each. A single dose of 5-FU (100 mg kg^−1^ body weight) or water only (negative control) was injected intraperitoneally into test mice. Starting 24 h after 5-FU administration, mice were force-fed cucumber extracts (10 or 50 mg kg^−1^ body weight) or *Dioscorea* extracts (10 mg kg^−1^ body weight), or given subcutaneous injections of rmG-CSF (5 *μ*g kg^−1^ body weight) for 5 consecutive days, once every day. Animals were sacrificed 24 h after administration of the final dose of *Dioscorea* or G-CSF.

### 2.7. Colony-Forming Unit Assay

The colony-forming activities of CFU-GEMM, BFU-E and CFU-GM in bone marrow cells (BMCs) were evaluated by culturing mouse femur and tibia BMCs in methylcellulose medium (Methocult GF M3434, Stem Cell Technologies, Vancouver, CA, USA). Murine BMCs were diluted to a final cell density of 1 × 10^5^/mL in Iscove**'**s Modified Dulbecco**'**s Medium containing 2% fetal bovine serum. For duplicate cultures, 0.3 mL of cell suspension was added to 3 mL of M3434 medium, gently vortexed and allowed to stand for 5–10 min to dissipate air bubbles. Aliquots of 1.1 mL cell suspension were dispensed into 35-mm culture dishes using an 18-G needle-attached syringe. The culture dishes were rotated gently to spread the methylcellulose gel evenly onto the substratum. Cell cultures were incubated for 12 days at 37°C with 5% CO_2_ in a humidified (≥95%) incubator, and the cell colonies and colony types were evaluated and counted under an inverted microscope (IX70; Olympus).

### 2.8. Sugar Composition and Linkage Analysis

Sugar composition was determined by gas chromatography mass spectroscopy (GC-MS). GC–MS was carried out using a HP-5MS fused silica capillary column (30 m** × **0.25 mm i.d., Hewlett Packard) at 60°C on a gas chromatograph (HP6890, Hewlett Packard) connected to an mass selective detector (HP5973, Hewlett Packard). The heat profiles for analyses of TMS and FAMEs were 60°C for 1 min, increasing to 140°C at 25°C min^−1^ and 200°C at 5°C min^−1^, and finally to 300°C at 10°C min^−1^. For partially methylated aditol acetate derivatives, the oven heat profile was 60°C for 1 min before increasing to 290°C at 8°C min^−1^, and finally to 300°C at a rate of 10°C min^−1^. Peaks were analyzed by GC-MS and compared with an established database. Arabitol derivative was used as an internal standard.

The GC-MS analysis of *Dioscorea* tuber extract was performed by methanolysis with 0.5 M methanolic/HCl at 80°C for 16 h, and trimethylsilyation with the Sylon HTP (HMDS/TMCS/pyridine, 3 : 1 : 9) trimethylsilyation reagent (Supelco). The final trimethylsilyated (TMS) derivatives were kept in *n*-hexane for GC-MS analysis [1]. Hakomori methylation analysis was used to examine carbohydrate linkage [2]. The test polysaccharide was pre-O-methylated with methyl iodide and dimethylsulfoxide anion in dimethylsulfoxide, and then hydrolyzed by 2 M trifluoroacetic acid at 120°C for 2 h. The solvent was evaporated by a stream of compressed air, and the residue was reduced with 0.45 M NaBD_4_ in 1 M NH_4_OH for 2 h at room temperature. The reaction was quenched with 100% acetic acid and co-evaporated with 10% acetic acid/methanol. The residue was peracetylated with acetic anhydride at 100°C for 1 h. After CHCl_3_/H_2_O partition, the sample was suspended in *n*-hexane, and finally analyzed by GC-MS. The analytical methods for GC-MS followed the parameters as reported previously [22, 23].

### 2.9. Statistical Analysis

All data are expressed as means ± SD. Differences between treatments were determined using SPSS statistics 17.0 with Student's *t*-test (two-tailed) or ANOVA. *P* < .05 was considered statistically significant. Levels of confidence are indicated as: *P < .05; **P < .01 and ***P < .001.

## 3. Results

### 3.1. DsCE-II Stimulates Growth of Murine Splenocytes in Primary Culture

**Figure 2:**
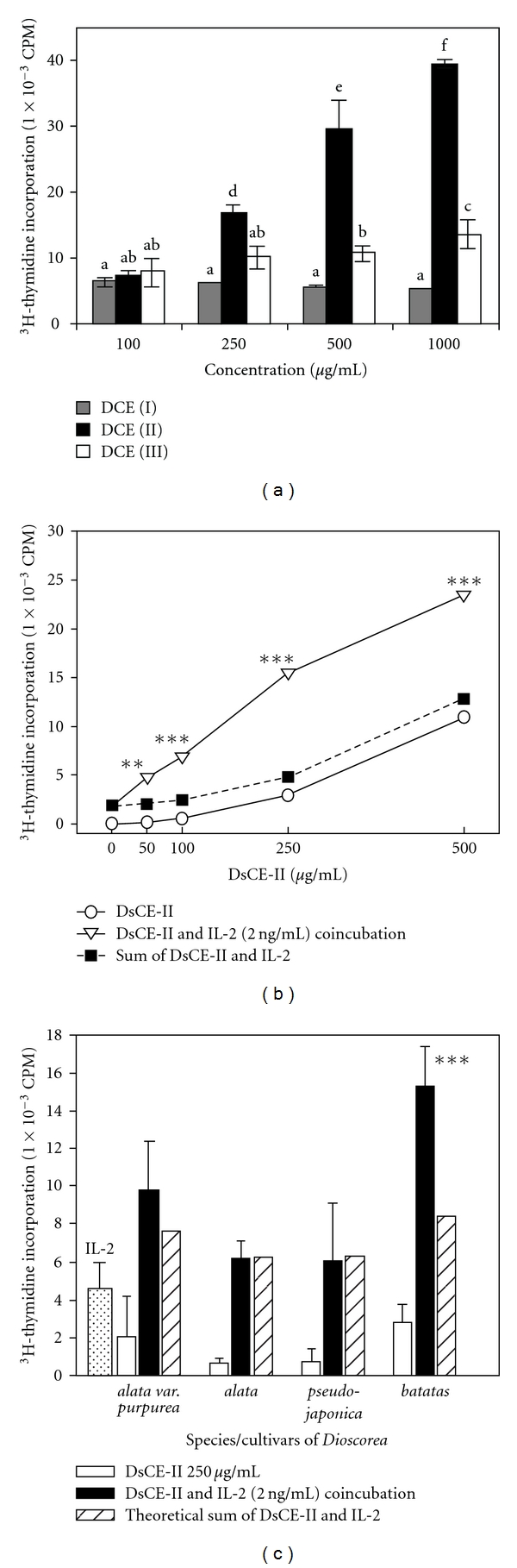
Effect of ethanol-partitioned DsCE fractions of *Dioscorea* tuber extract on the proliferation of murine splenocytes, alone or in combination with IL-2. (a) Splenocytes isolated from BALB/c mice were incubated with varying amounts (0–1000 *μ*g mL^−1^) of DsCE-I, -II or -III. Bars with different alphabets represent significant differences between treatments. (b) Splenocytes were incubated with increasing amounts (0–500 *μ*g mL^−1^) of DsCE-II of *D. batatas* Decne, in combination with 2 ng mL^−1^ IL-2 in culture medium. The cpm value for splenocytes treated with 2 ng mL^−1^ IL-2 alone was determined at 1,864 ± 272. Open circles denote the growth activity of splenocytes treated with DsCE-II; inverted open triangles show the experimentally observed stimulatory activity when DsCE-II and IL-2 were added together to test cells; filled squares indicate the theoretical total activity determined by adding the individually obtained activities of DsCE-II and IL-2 as a sum. This result indicates that DsCE-II and IL-2 have a synergistic effect rather than an additive effect. CPM values that differed significantly from theoretical sum are marked. (c) The specific DsCE-II fractions extracted from the tuber of four different species/cultivars of *Dioscorea* spp. plants were tested alone or in combination with IL-2 for their abilities to stimulate splenocyte cell proliferation in culture. After treatment for 48 h, test cells were then labeled with ^3^H-thymidine and harvested for assay as described in the ‘Methods' section. The data represent the mean ± SD of triplicate cell culture samples. Two other independent experiments were repeated and showed similar results. Significant difference (***P < .001) between the group of DsCE-II with IL-2 coincubation and theoretic sum. **P < .01.

In the absence of pre-treatment with known mitogens (e.g., phytohemagglutinin, concanavalin A or cytokines), the DsCE-II fraction of *D. batatas* increased the proliferation of murine splenocytes in a dose-dependent manner (*P* < .001) (Figure 2(a)). This suggests that specific phytocompounds in DsCE-II could confer specific immunomodulatory activities resulting in splenocyte proliferation. In contrast, DsCE-I failed to induce cell proliferation, and DsCE-III only induced slight proliferation. In the linear range (100–500 *μ*g mL^−1^) of DsCE-II stimulation, unless otherwise indicated, we chose 250 *μ*g mL^−1^ for subsequent experiments.

The crude water extract and the derived DsCE-I, -II and -III subfractions of *D. batatas* were further studied for their possible cytotoxicity on cell proliferation, using human skin fibroblast (CCD966SK), human hepatoma (Hep G2 and Huh 7) and human mammary carcinoma (MCF-7) cell lines in standard MTT assays [24]. The *Dioscorea* extracts and subfractions exhibited no detectable cytotoxic activity, that is, between 96 and 105% cell viability was observed for test cell samples as compared with control samples (data not shown).

### 3.2. Effect of DsCE-II and IL-2 Co-Treatment on Splenocyte Proliferation

We incubated splenocytes with mixtures of increasing amounts of DsCE-II extract from *D. batatas* and a fixed concentration (2 ng mL^−1^) of interleukin (IL)-2. Even at low concentrations (50 *μ*g mL^−1^), DsCE-II effectively stimulated the splenocyte growth induced by IL-2 (Figure 2(b)). The addition of 250 *μ*g mL^−1^ DsCE-II and 2 ng mL^−1^ IL-2 together resulted in a 3.2-fold higher cell proliferation activity than the sum of stimulatory levels of DsCE-II and IL-2 individually (square symbol in Figure 2(b)). Thus, IL-2 and DsCE-II acted in concert to synergistically enhance splenocyte proliferation. A similar synergistic proliferation was also observed when a fixed concentration (250 *μ*g mL^−1^) of DsCE-II was combined with various doses of IL-2 up to 5 ng mL^−1^, in cell proliferation assays (data not shown). In parallel, plant extract samples from an Orchidaceae herb, *Anoectochilus formosanus*, were similarly tested for their effect on splenocyte proliferation. No synergy was detected (data not shown). Taken together, our results demonstrate that IL-2 and DsCE-II can synergistically enhance the proliferation of mouse splenocytes *ex vivo*.

### 3.3. Co-Stimulatory Activities in Four Varieties of Dioscorea Plants

To detect whether the cell proliferative and synergistic effect above was specific to *D. batatas*, DsCE-II extracts from four species/cultivars of *Dioscorea* were tested for their effects on splenocyte proliferation and their ability to enhance IL-2-mediated cell growth. DsCE-II fraction from *D. batatas* had a more significant effect on splenocyte proliferation than the DsCE-II fractions from the other species/cultivars; this observation was also true with respect to the synergistic effects of IL-2 (*P* < .001) (Figure 2(c)). These results suggest that the stimulatory effect on splenocyte proliferation exhibited by DsCE-II plant species/cultivar is specific and demonstrate the importance of using specific plant species and standardized preparation(s) of herbal extracts for therapies.

### 3.4. DsCE-II Promotes Regeneration of Murine BMCs and Splenocytes in 5-FU-Treated Mice

**Figure 3:**
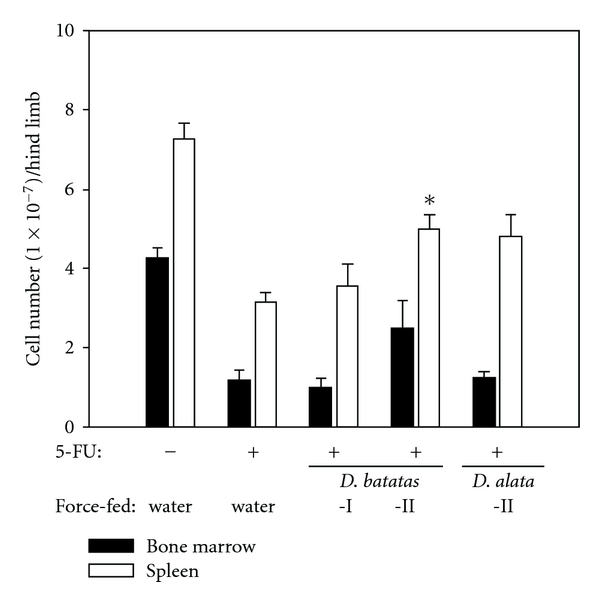
Fractionated tissue extracts of *Dioscorea* tuber effectively help to regenerate or recover test cells of bone marrow and spleen tissues of 5-FU-treated BALB/c mice. Mice were pre-treated with 5-FU at 100 mg kg^−1^ body weight; 24 h later, test mice (three mice per group) were force-fed for five consecutive days with 0 or 10 mg kg**^−1^** body weight of *D. batatas* Decne tuber extracts (DsCE-I or DsCE-II). Experimental data are expressed as mean ± SD. Filled squares indicate BMCs; open squares depict splenocytes. Significant difference (*P < .05) when compared with 5-FU treatment control. An independent experiment was repeated and similar results obtained.

To assess the possible immunomodulatory effect of *Dioscorea* extracts *in vivo*, we force-fed *Dioscorea* extracts to BALB/c mice. Oral feeding was chosen because in many Asian cultures, *Dioscorea* is consumed as a constituent of tonic teas or soups. The effect of DsCE-II on spleen or BMC numbers of healthy normal mice was negligible. We therefore assessed the effects of *Dioscorea* on immune-impaired mice. Mice were first treated for 1 day with the chemotherapeutic drug 5-FU. 5-FU is strongly cytotoxic to mitotic active tumor cells but also has profound side effects, including toxic effects on the lymphoid, myeloid and other hematopoietic cells in bone marrow tissue [19]. To determine the appropriate dose of 5-FU necessary to confer an effective but recoverable level of cytotoxicity, mice were treated with 50, 100 or 150 mg kg^−1^ body weight of 5-FU. In total, 100 mg kg^−1^ body weight 5-FU induced a significant decrease (60–80% of the level of untreated controls) in both bone marrow and spleen cell populations (data not shown): therefore, in the following experiments, mice were treated with 100 mg kg^−1^ body weight of 5-FU, and then fed with various *Dioscorea* tuber extracts or water only (vehicle control). We treated mice with 5-FU for 1 day and then began force-feeding them with DsCE-I or DsCE-II (10 mg kg^−1^ body weight) from *D. batatas* for 5 consecutive days. After six days, the average BMC count in untreated mouse hind limbs was (4.22 ± 0.27) × 10^7^ cells (100%) (Figure 3, black bar). The average BMC count for cohort mice treated with 5-FU alone or 5-FU and DsCE-II of *D. batatas* was 1.17 ± 0.25 × 10^7^ (27.7% to controls) and 2.46 ± 0.68 × 10^7^ (58.3% to controls), respectively. In other words, treatment with DsCE-II approximately doubled the number of BMCs in 5-FU-treated mice, recovering BMCs to half the number in healthy mice. DsCE-I, however, conferred no effect on BMC regeneration. A similar DsCE-II protective effect was also observed for the regeneration of splenocyte cell populations from the same set of 5-FU-treated mice (Figure 3). Here, the splenocyte cell numbers were 7.25 ± 0.41 × 10^7^ (untreated, no 5-FU and DsCE-II, 100%), 3.13 ± 0.21 × 10^7^ (5-FU treatment only, 43.2%), 3.52 ± 0.58 × 10^7^ (5-FU treatment plus DsCE-I, 48.6%), and 4.98 ± 0.37 × 10^7^ (5-FU treatment plus DsCE-II, 68.7%). An approximate 1.6-fold increase in splenocyte population was found in the 5-FU-treated mice given DsCE-II *D. batatas*, as compared with 5-FU-treated mice given water only (*P* < .05) (Figure 3).

### 3.5. DsCE-II Treatment Restores Specific BMC Population in 5-FU-Treated Mice

To further evaluate the effect of DsCE-II on BMCs *in vivo*, we analyzed the effect of DsCE-II on the nucleated cell populations [21] in bone marrow tissue of 5-FU-treated mice. Approximately 79% of the nucleated cell populations were recovered in the 5-FU-treated and DsCE-II-fed mice as compared with the untreated control mice (100%) (data not shown). This represented *∼*1.83-fold increase in nucleated BMC count over the *∼*43% level obtained from the 5-FU and water-treated mice. In comparison, feeding with extracts of an unrelated control plant, (cucumber extract) conferred a marginal (*∼*51%) recovery effect on BMCs in test mice, which shows that the effect of the DsCE-II fraction was specific to plant tissue. This result is also consistent with that shown in Figure 3.

### 3.6. DsCE-II Preferentially Stimulates Regeneration of CFU-GM Cells in Bone Marrow

Nucleated BMCs from control mice (without 5-FU), as well as nucleated BMCs from mice pretreated with 5-FU and then force-fed water, cucumber extract as negative controls, or DsCE-I or -II extracts were subsequently cultured and quantitatively assayed for growth of specific colony-forming units (CFU) of progenitor cells: CFU-GEMM, CFU-GM and BFU-E. The number of CFU grown from untreated mice in methylcellulose gel for the three test cell types was consistent with those of previous studies (Table 1) [19, 20]. The CFU numbers for CFU-GM, BFU-E and CFU-GEMM cells in 5-FU-treated mice were substantially reduced, to 27.6, 10.9 and 8.2%, respectively, of numbers in control mice (Table 1). Of the mice force-fed with DsCE-II, populations of CFU-GM recovered to 67.3% (*P* = .014): CFU-GEMM and BFU-E cell lineages recovered substantially less, to 21.4% (*P* = .395) and 11.9% (*P* = .99), respectively (Table 1). DsCE-II treatment produced *∼*2.4-fold (27.6–67.3%) and 2-fold (10.9–21.4%) recovery of GM and BFU-E cells, respectively, in comparison to the same cells in the mice treated with 5-FU and water. Slight reduction or little change (8.2–11.9% = 3.7% of total CFU) was detected for GEMM cells in DsCE-II treated mice (Table 1). We observed much less effect on the recovery of the three cell types in the bone marrow tissues of 5-FU plus DsCE-I-treated mice in comparison with 5-FU plus DsCE-II-treated mice (Table 1). This trend of results was consistent in two other independently repeated experiments.

We then tested the cytokine G-CSF, a recombinant protein drug for assisting the recovery of leukocytes in bone marrow chemotherapy patients as a ‘positive' control of the *in vivo* study. Subcutaneous administration of 5 *μ*g kg^−1^ recombinant murine G-CSF (rmG-CSF) effectively recovered test cells in 5-FU-treated mice to 78.9% of control levels (no 5-FU) (Table 1). It had a modest effect on GEMM cells (25%) and little effect (5.6%) on BFU-E cells. The degree of recovery of CFU-GM cells in 5-FU and DsCE-II-treated mice (67.3%), and 5-FU and G-CSF-treated mice (78.9%) was quite similar, 2.44- and 2.86-fold, respectively, higher than that of the mice given 5-FU plus water (27.6%). Mice given 5-FU cucumber as a control, showed little or no regeneration of CFU-GM, BFU-E or GEMM cells. The data in Table 1 were obtained from an average of results from two independent experiments. Furthermore, two other experiments were carried out, and the effect of DsCE-II and G-CSF on the three progenitor cell lineages was similar. These results suggest that DSCE-II is highly specific in its action on different bone marrow progenitor cell types.

Since hematopoietic progenitor cells can also be translocated from the spleen into bone marrow tissue [25], we surgically removed the spleens of test mice, and then subjected the mice to the tests in Table 1. We observed 8.2 ± 0.4, 27.4 ± 3.5 and 100 ± 9.2% of CFU-GM cells in bone marrow tissues of mice without spleens treated with 5-FU and water or DsCE-II, or 5-FU alone, respectively (complete set of data not shown). Thus, we observed a 3-fold increase in CFU-GM cell recovery in mice given DsCE-II as compared with those given water even in animals with spleens removed, although the capacity for CFU-GM recovery was considerably lower than in mice with intact, healthy spleens (Table 1).

This result supports the finding that recovery of specific BMC populations can be directly affected by oral feeding with DsCE-II.

### 3.7. Fractionation and Characterization DsCE-II Components

**Figure 4:**
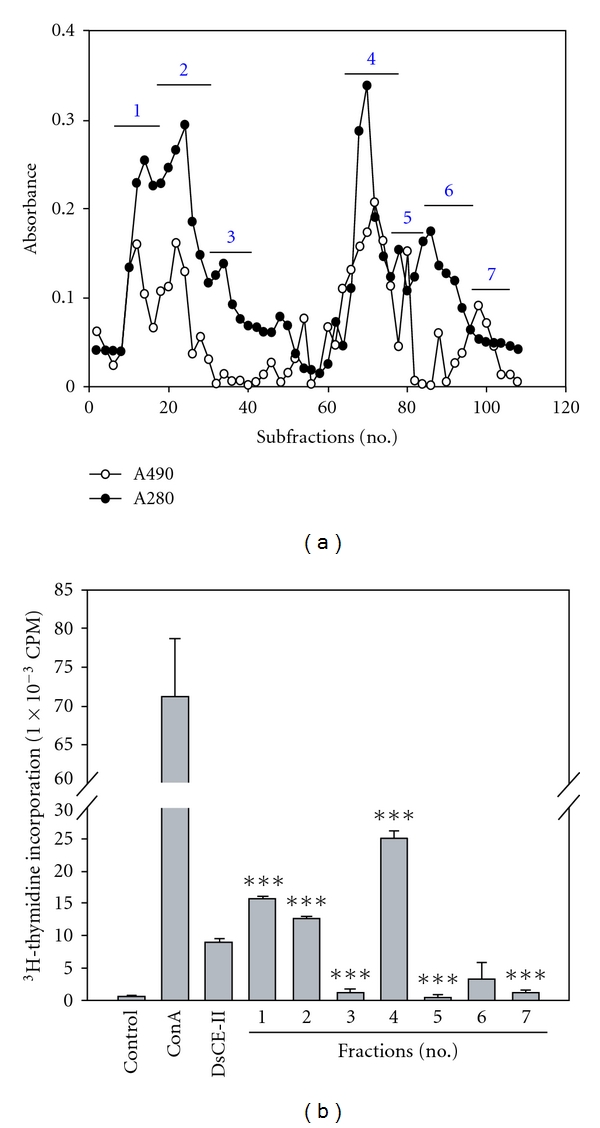
Fractionation and characterization of phytocompound components of DsCE-II. (a) Anion exchange column chromatography of DsCE-II. DsCE-II fraction of *D. batatas* Decne was separated into seven fractions [1–7] by use of a Q Sepharose ion exchange column and eluted with 0–0.75 M NaCl salt gradient in 5 mM phosphate buffer (pH 7.0). Absorbance at 280 nm was recorded for polypeptides, and absorbance at 490 nm of each fraction was determined by use of the phenol-sulfuric acid method for the content of saccharides as described in Section 2. (b) Bioactivity assay of subfractionated components of DsCE-II. The bioactivity of each subfraction at (250 *μ*g mL**^−1^**) was measured by the murine splenocyte proliferation assay as described in Section 2. Negative control, Con A (positive control, 1 *μ*g mL**^−1^**), and the original un-fractionated DsCE-II extract (250 *μ*g mL**^−1^**) were assayed in parallel. Data represent the mean ± SD of triplicate cell culture samples. A repeated experiment showed similar results. Significant differences (***P < .001) between fractionated samples and the original un-fractionated DsCE-II.

The bioactive DsCE-II from *D. batatas* tuber extract was further analyzed for its phytocompound components by anion exchange column chromatography. By filtering through a Q Sepharose Fast Flow ion exchange column (Pharmacia), seven subfractions (fractions #1–7, Figure 4(a)) showing major absorbance peaks at 280 and 490 nm for polypeptides and saccharides, respectively, were collected and subjected to bioactivity testing using mouse splenocyte proliferation enhancement assay. Fraction #4 conferred a level of specific activity *∼*3-fold higher than that of the unfractionated DsCE-II, *∼*2-fold higher activity than that of fractions #1 and #2, and an 8- to 50-fold higher activity than that of fractions #3, #5, #6 and #7 (Figure 4(b)). Fraction #4 contained both polysaccharides and polypeptide components, and SDS-PAGE analysis revealed that it contained a dominant protein band, representing probably the major tuber storage protein of *Dioscorea*, dioscorin, as confirmed by N-terminal protein sequencing. Bioactivity analysis further showed that the increase in splenocyte proliferation by fraction #4 was unlikely to be caused by protein substances that might exist in the fraction, because exhaustive trypsinization of fraction #4 did not significantly (≤12%) reduce the resulting bioactivity in the splenocyte proliferation assay (data not shown). Additional experiments revealed that the ability of DsCE-II to stimulate splenocyte proliferation was totally inhibited by lipid A inhibitor (polymyxin B; P-1004, Sigma, MO, USA), but this activity had little or no effect on the synergy between IL-2 and DsCE-II (data not shown).

**Figure 5:**
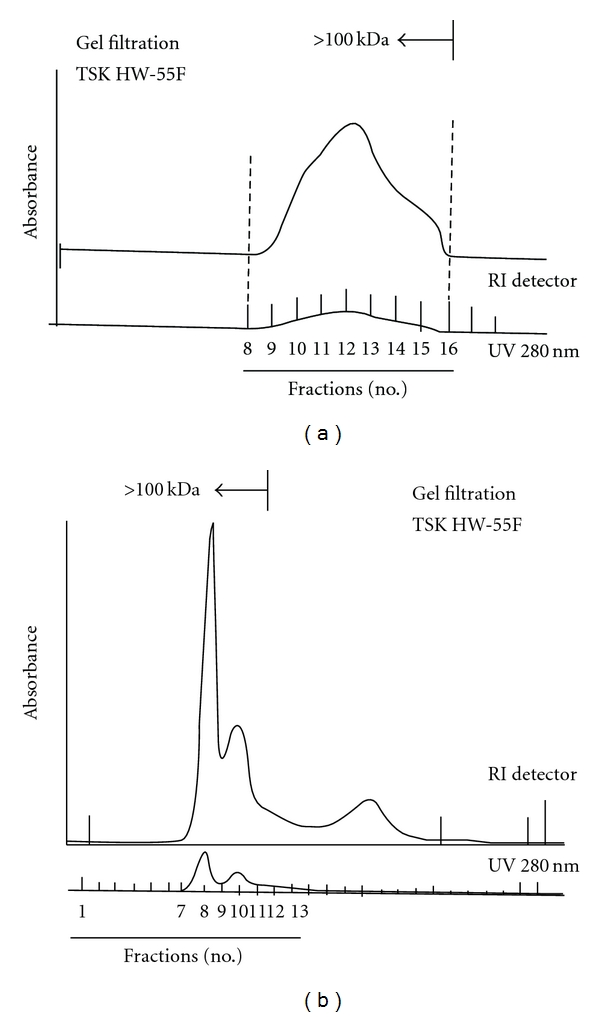
TSK HW-55F gel filtration and profiling analysis of polysaccharides of DsCE-I (a) and -II (b) fractions from *D. batatas*.

Since the isolated DsCE-II material dissolved in aqueous solution was viscous and gluey, this characteristic strongly influenced the chromatographic flow and contributed to the asymmetrical profile detected in fractionation by size-exclusion chromatography using a TSK HW-55F column (Figure 5(a)). A great majority of the DsCE-II molecules are polysaccharides with an apparent molecular mass of >100 kDa, as determined by RI detector and the phenol-sulfuric acid method, with the use of several dextrans, including blue dextran (2000 kDa), 500, 70 and 10 kDa dextrans, as molecular mass standards. DsCE-II may also contain a very low level of high molecular weight polypeptides because some absorbance at 280 nm was detected. In parallel, we characterized the chemical content in DsCE-I, as referencing information to that of DsCE-II, because DsCE-I showed no detectable activity compared with DsCE-II (Figure 2(a)). As shown in Figure 5(b), symmetrical peaks exhibiting a relatively high absorbance in RI detector as well as at UV_280_ in size exclusion column were obtained for DsCE-I, a watery, not viscous extract. Based on the sugar composition and linkage analysis by GC-MS, we further demonstrated that DsCE-I and DsCE-II can be readily distinguished by their difference in composition of *β*-1,4-linkage glucose versus *β*-1,4-linkage mannose (Table 2). We thus suggest that the major immunomodulatory constituent(s) in DsCE-II is likely contributed by the high content of mannose polymer (≥64%). Further analyses are needed for gaining more insights into the specific biochemical identities in the DsCE-II and the fraction #4 components of the *D. batatas* tuber extracts.

## 4. Discussion

This study provides scientific evidence for the immunomodulatory activity of tuber tissues of *Dioscorea* spp. The *Dioscorea* tuber is widely used as a functional food or phytodrug in several Eastern Asian cultures. It is usually served either as a fresh vegetable or in the form of stewed tubers in tonic soups. Tuber slices are also an essential component in many TCM recipes. It is striking that in ancient Chinese herbal medicinal literature such as Ben-Cao-Kong-Mu [13], Chinese medical doctors often relate the significant positive homeostatic/immune-regulatory effects of *Dioscorea* tuber specifically to the species *D. batatas* Decne., but not other *Dioscorea* species. Our present study supports this historical claim. In addition, in this study we demonstrate that specific ingredients in DsCE-II, a fractionated tuber extract of *D. batatas*, not only stimulated immune cell proliferation, but also aided the regeneration of bone marrow and spleen in 5-FU-pretreated animals.

The ability of *Dioscorea* to modulate immune signal transduction in the regulation of cytokine expression has recently been studied. A specific glycoprotein isolated from *D. batatas* has been shown to inhibit the expression of IL-4 and IL-10 through modulation of GATA3, STAT6 and p44/42 or p38 MAPK in primary mouse lymphocytes [26]. And diosgenin, a steroidal sapogenin of *Dioscorea*, was found to enhance antigen-specific expression of IgG2a and IFN-*γ*, which may mediate the up-regulation of Th1 differentiation [27]. These *ex vivo* studies demonstrate that phytocompounds from *Dioscorea* can modulate specific immune system activities via regulation of specific cytokine and chemokine networks. Our present study showed that co-treatment with DsCE-II and cytokine IL-2 can promote splenocyte proliferation. The dose-response profiles for DsCE-II, both alone and in combination with IL-2, imply that components of DsCE-II may interact with each other or, more interestingly, with IL-2, perhaps synergistically. The potential for synergy between different components in herbal medicine mixtures is a fundamental concept in TCM [28]. One recent study [29] demonstrated a plausible molecular basis for the synergistic anti-tumor effect of two components of a TCM formulation. It will be interesting to determine in future studies whether DsCE-II functions synergistically or merely additively with IL-2 or other cytokines in splenocytes.

Oral administration of a TCM herbal extract has been shown to induce proliferation of erythroid progenitor cells in mice with 5-FU-induced anemia [30]. In this study, we found that mice which were fed with a *Dioscorea* extract recovered damaged bone marrow progenitor cell populations that had been depleted by large doses of 5-FU. The improved recovery of these specific immune cells in 5-FU plus DsCE-II-treated mice in comparison with mice treated with 5-FU alone, was highly consistent across three independent experiments (Figure 3). In order to evaluate the underlying cellular mechanism(s), we analyzed in more detail the response of different hematopoietic progenitor cell types in mouse bone marrow to DsCE-II treatment. The recovery rate in 5-FU-treated mice for CFU-GM cells mediated by DsCE-II (*D. batatas*) was similar to that mediated by granulocyte colony-stimulating factor (G-CSF), implying a potential clinical application of DsCE-II. G-CSF is a well-recognized cytokine that can reduce the incidence of febrile neutropenia in chemotherapy and is used as a supportive therapeutic agent for patients undergoing bone marrow transplantation [31]. A recent report shows that G-CSF can shift the balance to granulopoiesis during myeloid lineage specification by activating STAT3 and stimulating SHP2 (Src homology region 2 domain-containing phosphatase 2) phosphorylation in marrow progenitor cells [32]. Therefore, it will be important to evaluate in future studies whether any of the components in DsCE-II directly activate STAT3 and SHP2 phosphorylation to enhance CFU-GM or indirectly enhance the expression of G-CSF, or whether other mechanisms are responsible for the regeneration of specific BMCs.

**Figure 6:**
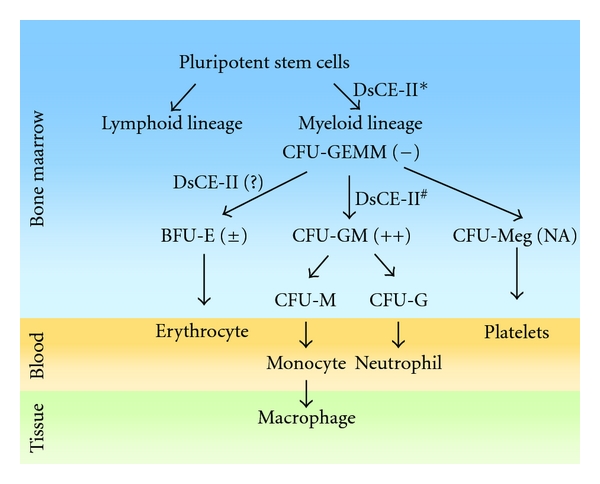
Hypothetical diagram for the effect of *Dioscorea* extract (DsCE-II) on regeneration of hematopoietic progenitors in bone marrow. −, no effect; ±, slight enhancement; ++, substantially increased; and NA, not analyzed. See legend to Table 1 for abbreviations for cell lineages. (DsCE-II*, bioactivity of DsCE-II on CFU-GEMM cells were tested and no effect was observed; DsCE-II^#^, DsCE-II greatly enhanced the level of CFU-GM cells).

Figure 6 shows our working hypothesis for the action mode specificity of DsCE-II in regenerating or promoting the growth of BMCs. Chemotherapy patients are often further treated with G-CSF to recover their immune systems. Our present study suggests that *Dioscorea* spp. may provide a much less costly, alternative approach. Since DsCE-II is an ethanol-partitioned fraction of an easily prepared, cold-water extraction of fresh *D. batatas* plant tuber, it could be readily made available in tablet or fresh paste form as a food supplement. The possibility of oral ingestion of *Dioscorea* tuber extract is especially attractive, because many Asian people have already routinely taken orally the uncooked or cooked tuber of this plant as a health food, for many cases over hundreds of years. To our best knowledge, no toxic effects from the oral ingestion of fresh *Dioscorea* tuber have been reported. In our preliminary studies (data not shown), we observed that the derived DSCE-II materials were safe in test mice, in terms of normal animal physiology, behavior and general toxicity parameters. However, for routine medicinal use of DsCE-II, clinical and toxicological studies are required in future studies.

In this study, the administration of DsCE-II phytocompounds via the oral route meant the gastrointestinal (GI) tract was the initial tissue target for *in situ* interaction, with the effects of treatment then observed at distant sites (i.e., bone marrow and spleen). Furthermore, the phytocompound(s) responsible for these bio-activities had a high molecular weight (≥100 kDa) and are most likely polysaccharide in nature. Therefore, we postulate that there are two likely routes by which DsCE-II molecules reach their target tissues, either: (i) the high-molecular-weight DsCE-II molecules are processed into smaller molecules and absorbed into GI tract tissues and then into the blood stream or alternatively (ii) DsCE-II molecules interact directly, in high molecular weight form, with the GI tissue. In the first case, oligosaccharides or other low-molecular-weight molecules that result from digestion could be taken up by intestinal tissues and absorbed into the circulation conferring their action(s) on cells in the blood or at distant target sites. Lysosomal *α*-mannosidase, for example, partially digests specific oligomannose moieties for antigen processing via CD1e by T-cells [33]. In the second case, a direct interaction between high-molecular-weight phytocompounds and GI tract tissues would have to occur; however, the possible mechanism(s) of such interaction is not obvious. Three recent findings may offer clues about such possibilities. First, it was reported that intestinal wall tissue is not totally “sealed” but instead contains intercellular paths that can expose various cell types (including dendritic cells and other immune-responsive cells) of the internal intestinal tissue to the nutrient contents of the GI tract, including macromolecules and even debris or pathogens [34, 35]. Furthermore, a high mannose-containing polysaccharide, with specific oligosaccharide sequence and structures that acts as a specific ligand for both DC-SIGN and DC-SIGN-receptor molecules in dendritic cells was identified. DC-SIGN and DC-SIGN-receptor molecules are two very important functional molecules of a key immune cell type that plays a central role in antigen presentation and other immune cell activities [36]. In addition, the mycobacterial antigens, the hexamannosylated phosphatidyl-myoinositols were recently reported to stimulate CD1b-restricted T-cells for antigen processing by CD1e [33]. Based on these and other reports on the role of specific polysaccharides as immunomodulators [36–41], we postulate that high-molecular-weight polysaccharides in DsCE-II act on specific target cell types in the GI tract (dendritic cells, intestinal epithelial cells, and T-cells) to mediate a cascade of immunoregulatory activities leading to the recovery of damaged cell populations following 5-FU or other chemical insults in the bone marrow, spleen or other immune cell systems.

In conclusion, we suggest that the *D. batatas* extract DsCE-II should be considered for further development as a dietary supplement alongside chemotherapy for cancer treatment, and the co-stimulatory effect of DsCE-II in combination with IL-2 may be useful in alternative cancer immunotherapy strategies.

## Funding

National Science and Technology Program for Agricultural Biotechnology (grant number 89AB704); an institutional grant from Academia Sinica; National Science Council Grant of Taiwan, ROC (NSC90-2811-B-001-023) and a National Science Council (NSC-97-2320-B-001-012-MY3).

## Figures and Tables

**Table 1:** Stimulatory effect of DsCE-II on regeneration of myeloid progenitor CFU-GM cells in bone marrow of 5-FU-treated mice.

Treatment	CFU number × 10^−2^ (percentage of control)		
	GM (%)	BFU-E (%)	GEMM (%)
–5-FU control	635.2 ± 42.1 (100)^a^	24.8 ± 6.3 (100)	29.2 ± 4.6 (100)
+5-FU H_2_O	175.5 ± 17.1 (27.6)	2.7 ± 0.3 (10.9)	2.4 ± 0.4 (8.2)
Veg	205.7 ± 17.8 (32.4)*	3.1 ± 0.7 (12.5)	2.2 ± 0.4 (7.5)
DsCE-I	263.3 ± 18.7 (41.5)	2.4 ± 0.8 (9.7)	3.4 ± 1.0 (11.6)
DsCE-II	427.4 ± 49.2 (67.3)**	5.3 ± 0.9 (21.4)	3.5 ± 0.5 (11.9)
G-CSF	501.6 ± 15.7 (78.9)**	1.4 ± 0.5 (5.6)	7.3 ± 1.4 (25.0)

Mice were treated with 100 mg kg**^−1^** body weight 5-FU, after 24 h mice were force-fed with water, Dioscorea extracts (DsCE-I and II of *D. batatas*, 10 mg kg**^−1^** body weight), and vegetable (cucumber juice, 10 mg kg**^−1^** body weight), or injected subcutaneously with G-CSF (5 *μ*g kg**^−1^** body weight) for five consecutivedays. BMCs were harvested and subjected to colony assay as described in the ‘Methods' section. Bone marrow of one hind limb (femur and tibia) was assessed for CFU number. Data are expressed as mean ± SD for a group of three mice. This experiment was performed three times giving analogous results. Results are presented with data pooled from two independent experiments. BFU-E: burst forming unit-erythroid; CFU:colony-forming unit, GEMM: granulocyte, erythrocyte, monocyte and megakaryocyte; GM: granulocyte and macrophage.

^a^Percentage of colony number over normal (no 5-FU) controls.

Significant difference when compared with 5-FU treatment control *P < .05; **P < .01.

**Table 2:** GC-MS sugar linkage analysis of DsCE-I and -II polysaccharides extracted from *D. batatas*.

Percentage of total carbohydrate	Sugar residue^a^						
	Ara	Rha	Fuc	Xyl	Man	Gal	Glc
DsCE-I	1.1	0.7	0.2	0.8	11.4	14.7	71.1
DsCE-II	4.2	0.5	0.1	0.7	64.4	13.3	16.6

^a^Sugar residues and compositions were analyzed as described in Section 2.
